# Optimal duration of medical expulsive therapy for lower ureteral stones: a critical evaluation

**DOI:** 10.1007/s00240-024-01548-5

**Published:** 2024-03-23

**Authors:** Erhan Erdoğan, Gamze Şimşek, Alper Aşık, Hikmet Yaşar, Cahit Şahin, Kemal Sarıca

**Affiliations:** 1 Department of Urology, Sancaktepe Sehit Prof. Dr. Ilhan Varank Research and Training Hospital, Istanbul, Turkey; 2 Department of Urology, Biruni University, Medical School, Istanbul, Turkey

**Keywords:** Stone expulsion, Tamsulosin, Ureteral stones, Duration

## Abstract

To evaluate the optimal duration of Medical Expulsive Therapy (MET) application for distal ureteric stones on a time period based manner. 89 patients with 5–10 mm distal ureter stones received tamsulosin (0.4 mg) for MET and diclofenac sodium (75 mg) for analgesia. Patients were evaluated once a week for 4 weeks. Radiologic stone passage was evaluated by kidney ureter bladder (KUB) and ultasonography where non-contrast computed tomography (NCCT) was also performed if needed. While 23 cases (28.4%) were SF after first week, 23 were SF (28.4%) after 2 weeks, 9 cases (11.1%) after 3 and lastly 7 cases (8.6%) became SF after four weeks. Nineteen (23.5%) cases were not SF after 4 weeks. A positive relationship was found between the time period elapsed for stone passage and ureteral wall thickness (UWT) along with the degree of hydronephrosis. In addition, mean number of renal colics and emergency department (ED) visits were found to be higher in patients passing stones in the 4th week along with the ones who could not despite MET. SFR for distal ureteric stones sizing 5–10 mm was higher within the first 3 weeks under MET application. Thus, waiting for a longer period of time may result in increased analgesic and unnecessary MET treatment with increased risk of emergency department visits and additional costs as well. We believe that other options could be considered in such cases who are not SF at the end of the first 3 weeks.

## Introduction

Urolithiasis is an important health problem with an increasing incidence in all parts of the world. The overall reported incidence is approximately 4% in the adult population where ureteral stones constitute 20% of them [1, 2]. Regarding the localization, approximately 70% of these stones seem to locate in the distal part of the ureter [3, 4].

Management of ureteral stones include either conservative [observation by pain control and hydration, medical expulsive therapy (MET)] or interventional approaches [antegrade or retrograde ureteroscopic lithotripsy, extracorporeal shock wave (ESWL) lithotripsy, laparoscopic or open surgery and ureterolithotomy] as the available treatment alternatives. Rational approach needs to be made based on the stone and patient related factors. Due to the risk of complications during interventional procedures (ureteral perforation, avulsion, and stenosis) and cost issues, observation along with MET is the treatment of choice for majority of ureteric stones sizing < 10 mm.

Regarding the chance for the spontaneous expulsion of distal ureteral calculi, stone size is the critical parameter where the likelihood for stones smaller than 4 mm is approximately 95%. This rate drops to 47% when the stone size is between 5 and 10 mm [5].

MET with alpha-blockers, calcium channel blockers, corticosteroids and phosphodiesterase-5 inhibitors (PDE5) has been identified as an effective treatment option in the medical management of distal ureteric stones to ease the spontaneous passage and reduce the number of colic attacks [6]. Of all the alpha-blockers used with this aim, tamsulosin has been the most commonly prescribed one. Main aim of this approach is to ensure smooth muscle relaxation without disrupting the physiologic ureteral peristalsis, relieve colic pain and eliminate edema as well as inflammation in the ureteral wall. As a result of these effects higher stone expulsion rates in a shorter time with less analgesic need have been anticipated [7]. However, despite the above mentioned effects of this medication confirmed so far, the issue is still under debate and some other trials reported contradictory outcomes [8]. Related with this issue, in their original randomized study including 1136 adults with ureteral colic Pickard et al. did not find a difference between active MET (Tamsulosin) and placebo with regard to spontaneous stone passage within 4 weeks [9].

Although the efficacy and safety of MET has been studied in various trials so far, the optimal duration of MET application for distal ureteric stones sizing 5–10 mm under observation is still unclear. An application period varying between 2 and 6 weeks period have been suggested by various authors particularly based on a study published in 1999 by Miler and Kane [10].However, an optimal duration based on the efficacy rates on a application time based manner has not been defined. EAU guidelines advises MET as a valuable medical therapeutic option (with strong recommendation) for distal ureteric stones however no discrete time period has been mentioned and/or recommended regarding the optimal duration with this aim [11].

In this study, we aimed to determine the optimal duration of MET in the medical treatment of distal ureteral stones sizing 5–10 mm.

## Patients and methods

Between January 2023 and June 2023, 89 patients with symptomatic, distal ureteral stones (detected between the lower border of the sacroiliac joint and the vesico-ureteral junction on non-contrast CT) sizing 5 to 10 mm were included in the study. The study protocol was approved by the local ethical committee (2023/98) and an informed consent form was obtained from all patients.

A total of 81 patients completed the study and 8 patients were lost to follow-up. 62 (76.5%) of the patients were male, 19 (23.4%) were female with an age range of 22–74 (mean; 43 ± 11) years. Patients with bilateral ureteral stones, multiple stones, urinary tract anomalies, lower urinary tract dysfunction, previous endoscopic or open ureteral surgery and patients under the age of 18 years were all excluded. All patients were evaluated with a detailed history, physical examination, laboratory and radiologic tests. In addition to size and the Hounsfield Unit (HU) value of the stone, degree of hydronephrosis, and the ureteral wall thickness (UWT) at the stone site were all determined/ measured on NCCT images.

In addition to sufficient hydration (2.5 L/day), all patients were treated with 0.4 mg tamsulosin once a day at night/oral, and 75 mg diclofenac potassium twice ( 2 × 1) a day/oral for pain relief. Patients were requested to record the total amount of water consumed daily on a chart and filter their urine to capture the stone passed, record the time of stone expulsion, the amount of analgesic required, treatment side effects, the number of renal colic, and the number of emergency department visits during the follow-up period.

Patients were called for weekly evaluation with urine analysis, KUB radiography for radiopaque, and urinary system ultrasonography or for non-opaque stones. A NCCT was also performed in cases with suspicion of stone passage either on KUB of sonographic examination. The total follow-up period was 4 weeks and if stone-free status could not be achieved during this conservative treatment period, alternative management options such as URS or ESWL were recommended.

While the primary aim of our current study was to evaluate the duration of stone-passage time within 4 weeks, secondary aim was to evaluate the rate of renal colic and emergency department visits along with the need for analgesia during this period.

The size of the study was assessed by using the G-power 3.1 program and possible correlation between the duration of stone passage and gender, age, stone size, HU, UWT and hydronephrosis parameters was evaluated by using Spearman flash analysis test. Ap value less than 0.05 was considered to be meaningful. Results were evaluated using Spearman correlation and Kruskal–Wallis tests.

## Results

Evaluation of the data obtained in our study revealed following findings; while there was no relationship between stone expulsion time and gender; a relationship was found between patient age, stone size, HU, UWT, hydronephrosis and stone expulsion time which was not statistically significant.

Regarding the time period for stone expulsion in our group, while 23 (28.4%) patients passed the stones within the first week follow-up, 23 (28.4%) patients passed within the second, 9 (11.1%) patients within the third week and lastly 7 (8.6%) patients passed within the fourth week period. 19 (23.5%) patients could not become stone-free after 4 weeks and were directed to alternative treatments such as URS and ESWL (Table 1).

**Table 1:** Patients’s demographic characteristics and time period based stone-free rates

	Stone free (week)				Non stone-free	*P*
	1st	2nd	3rd	4th		
Gender Male/female	16/7	18/5	6/3	6/1	16/3	0.204
Age (mean ± SD*)*	44.4 ± 12	38.3 ± 9	51.2 ± 15	45.2 ± 8	43.8 ± 7	*0.027*
Total (*n*)	23	23	9	7	19	
Stone-free rate (%*)*	28.4	28.4	11.1	8.6	23.5	

A statistically insignificant relationship was found between stone-passage period and the age, stone size and UWT parameters. Time period required for stone period tended to increase parallel to the age, stone size, and UWT values (*p* = 0.027, *p* = 0.043, *p* = 0.013, respectively) (Table 2).

**Table 2:** Evaluation of stone related parameters in both groups

	Stone free (week)				Non stone-free	*P*
	1st	2nd	3rd	4th		
Stone size (mm ± SD)	5.95 ± 1.02	6.3 ± 1.2	7 ± 1.4	6.57 ± 1.6	6.42 ± 1.5	0.043
Hounsfield Units (HU ± SD)	564 ± 331	746 ± 376	541 ± 282	869 ± 505	776 ± 298	0.906
Ureteral wall Thickness (mm ± SD)	2.09 ± 0.4	2.16 ± 0.9	2.38 ± 0.7	2.16 ± 0.6	2.08 ± 0.7	0.013

However, we could not demonstrate and statistically significant relationship between time period for stone-free status and gender, hydronephrosis and HU values.

Among the side effects of the alpha blocking agents applied, retrograde ejaculation in 2 (2.4%) patients, itching in 4 (4.9%) patients, hypotension in 2 (2.4%) patients, diarrhea in 1 (1.2%) patient, dizziness in 1 (1.2%) patient, and umbilical purpura in 1 (1.2%) were noted and treated conservatively. No significant difference was detected between the groups on this aspect (*p* = 0.143).

Mean number of visit to the emergency department ranged between 0 and 10. While 82.6% of those passing the stone within 1st week, 83.7% of those within the 2nd week, and 88.9% of those within the 3rd week had no or only once ED visit, 71.5% of the cases expulsing the stone within the 4th week visited the emergency room more at least two or two or more times. As shown in Table 3, the average number of emergency department visits was lower in cases passing the stones during the 3 weeks duration of management when compared to those who passed the stones with in the 4th week follow-up period as well as in those who did not pass any stones at all. Kruskal–Wallis (KW) multiple comparison test analysis of the data on this aspect clearly showed a significant difference between those who passed their stones in the first 3 weeks and those who could not pass during a 4 week follow-up period (*p* = 0.005) (Table 3).

**Table 3:** Comparative evaluation of the mean number of ED visits and renal colic attacks

Mean value ± SD	Stone-free (week)				Non stone-free	*P*
	1st	2nd	3rd	4th		
Mean no of visits to the emergency department	0.91 ± 0.9	1.09 ± 1.3	1.44 ± 1.1	3.0 ± 2.0	2.89 ± 2.2	0.005
Mean no of renal colic attack	1.65 ± 2.1	1.69 ± 1.4	1.88 ± 1.9	4.29 ± 2.8	2.53 ± 2.0	0.011

On the other hand, again mean number of renal colic attacks ranged between 0 and 10 in our group. These values were 0, 1, or 2 in 82.5% of the patients passing stones during the 1st week and in 78.2% of the cases passing during the 2nd week and lastly 77.7% in those being stone free within 3 weeks, respectively. Mean renal colic number was 3 or more in 57.9% of those who had passed the stone within the 4th week. Similar to ED visits again, average renal colic number was found to be lower in cases passing their stones spontaneously under MET when compared to those who passed their stones during the 4th week evaluation along with the ones who were not stone free despite these measures.

Again comparative evaluation of the values in all groups with Kruskal–Wallis (KW) multiple comparison test, demonstrated a significant difference in the number of renal colic between those who passed their stones in the first 3 weeks and those who could not pass in the 4th week. *p* = 0.011 (Table 3).

In other words, our data clearly shown that if MET therapy prolongs to 4 weeks period or more, a significant increase could be noted in the mean number of renal colic attacks and mean emergency department visits in these patients.To support this observation, of the 26 patients who could not pass their stones within the first 3 weeks period, only 26.9% of them did pass their stones further during 4th week follow-up and the remaining 73.1% of the cases required interventional removal due to the problems affecting the life quality of the patients.

## Discussion

The main findings of this study demonstrated a statistically significant difference with respect to the mean numbers of renal colic and emergency department visits between patients who passed the stones within the first 3 weeks of therapy and patients whom had the stones in the lower ureter despite MET application for three weeks. Of course a limited percent of these cases also passed the stones during 4 week follow-up period but our results clearly demonstrated that a significant increase in the number of renal colic and emergency department visits requiring higher amount of analgesic use could be noted in these cases without any further contributive effect of MET.

Based on findings and the well defined underlying mechanisms, MET aims to relieve colic pain, eliminate submucosal edema and inflammation in the ureteral wall which will in turn accelerate the spontaneous passage of stone(s) with higher expulsion rates in relatively shorter time period. Additional advantages of MET are reduction of mean number of renal colics, visit to emergency department and ultimately the treatment cost. The above mentioned advantages of these agents particularly in terms of shortening the time period for spontaneous stone expulsion have been well demonstrated so far. In their original study, Miller and Kane were able to demonstrate significantly shortermean duration of stone expulsion following MET application compared to control group cases [10]. It is clear that the time period need for spontaneous stone expulsion was longer prior to the clinical introduction of MET application. Regarding this issue however, although some randomized clinical studies have shown the safe and effective use of these agents to accelerate stone expulsion and limit the associated problems affecting the quality of life [12–15]; contradictory outcomes have also been reported in other studies which makes the issue still debatable. Last but least, the response rate to these medications in terms of limiting the extent of edema formed in the ureteric wall could vary from patient to patient due to certain organism related factors affecting the outcomes.

Despite the well definition of the dosage and application details for these agents reported so far, there is no standardized and well accepted time period mentioned in the available clinical guidelines for their use. Generally a time period of 2 to 6 weeks is being recommended particularly for distal ureteral stones sizing 5–10 mm. Regarding this issue again, in the only study published in 1999, a follow-up period of 6 weeks has been recommended as the optimal duration by Miller and Kane [10]. In other words, although the efficacy and safety of MET have been well evaluated with various trials, the optimal duration of MET application for distal ureteric stones sizing 5–10 mm under observation is still to be clarified. Thus, an optimal duration regarding the efficacy rates on an application time based manner seems to be certainly needed. Although, EAU guidelines advises MET as a valuable medical therapeutic option (with strong recommendation) for distal ureteric stones, no discrete time period has been mentioned and/or recommended regarding the optimal duration with this aim [11].

On the other hand, application of MET can cause some certain side effects including hypotension, vertigo, diarrhea, itching and somnolence. If the stone(s), do not pass successfully under this therapy, prolonged use of these agents during watchful waiting period may increase the risk of such complications which will affect the life quality of these patients considerably. In addition, long-term presence of the stone in the same portion of the ureter may result in increased obstruction and infective problems which will increase number of visits to emergency department, renal colic attacks. All these factors will in turn increase the total treatment costs [16].

In light of our current findings again, we may state that patients in whom the MET application seems to be unsuccessful after three weeks need to be directed to other stone removal procedures in an attempt to provide a good life quality, limit the risk of upper tract changes due to the varying degrees of obstruction and of course limit the total costs without any loss of time. In addition, the risk of side effects induced by MET will be limited if the medication could be stopped on time. Supporting this observation no significant difference was noted between cases directed to other treatment methods and the patients passing their stones earlier. Thus our findings indicate that it could be rational and appropriate to apply MET for 3 weeks period and terminate MET it if the stone(s) do not pass during this period. Plan for other minimal invasive stone removal procedures based on the possible problems mentioned above will be most logical approach which will allow the physicians inform their cases about the aforementioned stone related problems and make plan for stone removal on time. To our knowledge, our study is the first one evaluating the final outcomes of MET in terms of stone-free status on a time based manner which will contribute to identify the optimal duration this therapy.

Our study is not free of limitations. First of all our current serie is a small one with limited the number of patients included. However, taking the relative difficulty of close-follow-up as well as documentation in such patients, we believe that our group with a perfect follow-up documentation will be contributive enough to the existing unclarified data in current literature.

## Conclusions

In light of our findings, since the vast majority of the cases passed the stones during the first three weeks of MET therapy, we recommend that this duration would be appropriate in patients presenting with distal ureteral stones of 5–10 mm in size. Patients who could not become stone-free at the end of this period and who do not want to wait under medication for a long-time period might be planned for alternative treatment options to limit the incidence of distressing colic attacks, ED referrals, and also possible side effects of the MET agents applied.

## Data Availability

The data supporting the findings of this study are available on request from the corresponding author.
